# Can autophagy enhance crop resilience to environmental stress?

**DOI:** 10.1098/rstb.2024.0245

**Published:** 2025-05-29

**Authors:** William Agbemafle, Vishadinie Jayasinghe, Diane C. Bassham

**Affiliations:** ^1^ Department of Genetics, Development and Cell Biology, Iowa State University, Ames, IA 50011, USA

**Keywords:** autophagy, stress response, crop improvement, autophagy manipulation

## Abstract

Climate change imposes abiotic stress on plants, significantly threatening global agriculture and food security. This indicates a need to apply our understanding of plant stress responses to improve crop resilience to these threats. Stress damages critical cellular components such as mitochondria, chloroplasts and the endoplasmic reticulum. Left unmitigated, abiotic stress can lead to cell death, which typically decreases overall plant health and productivity. Autophagy is a catabolic process that maintains cellular homeostasis by degrading and recycling damaged and dysfunctional cell components and organelles. Importantly, autophagy promotes plant tolerance to a wide range of environmental stresses, and manipulation of autophagy may lead to improved stress resilience in crops. Here, we discuss recent advances in our understanding of how autophagy affects abiotic stress resistance. We discuss the function of autophagy in different abiotic stresses (including nutrient stress, salt stress, drought, heat, cold, hypoxia, light stress and combined stresses) and provide insights from functional and genome-wide transcriptomic studies. We also evaluate the potential to enhance crop survival and productivity in suboptimal environmental conditions by activating autophagy, emphasizing the importance of targeted manipulation of key genes involved in the autophagy pathway.

This article is part of the theme issue ‘Crops under stress: can we mitigate the impacts of climate change on agriculture and launch the ‘Resilience Revolution’?’.

## 1. Introduction

**Figure 1.**
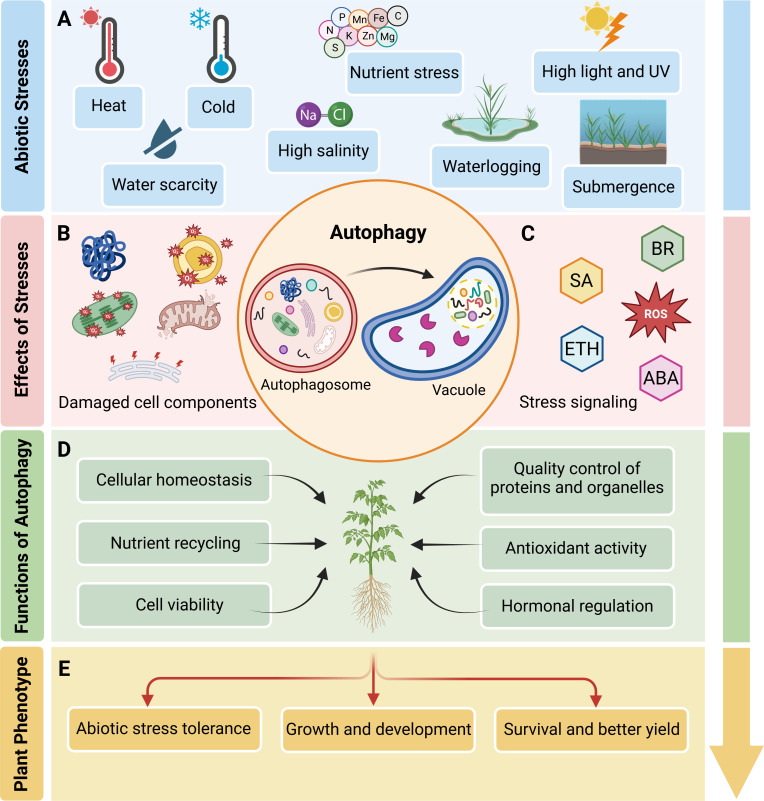
Role of autophagy in plant responses to abiotic stresses. (A) Plants experience a range of abiotic stresses, including heat, cold, water scarcity, high salinity, nutrient deficiency, excessive light, UV radiation, waterlogging and submergence, all of which ultimately affect plant growth and development. (B) Abiotic stresses can cause damage to cellular components, such as membranes, proteins and organelles, leading to disruptions in normal physiological functions. (C) In response to abiotic stresses, plants activate stress signalling pathways including those mediated by brassinosteroids (BR), abscisic acid (ABA), salicylic acid (SA), ethylene (ETH) and reactive oxygen species (ROS), that subsequently activate the autophagy pathway. (D) Autophagy aids in managing cellular damage and maintaining homeostasis by facilitating the degradation and recycling of damaged cell components. (E) This process ultimately enhances plant survival, increases tolerance of abiotic stresses and improves growth, development and yield.

Plants frequently encounter various stresses in the environment that negatively impact their productivity, growth and development [1]. To increase survival, plants activate intricate mechanisms to maintain cellular homeostasis during stress. Autophagy is a vacuolar degradation process that removes and recycles undesired cytoplasmic materials such as damaged organelles and misfolded proteins [2]. Autophagy performs housekeeping roles under non-stressful conditions to facilitate normal plant development. In response to stresses such as drought, starvation and hypoxia, autophagy activity increases to enhance plant survival [3] (figure 1).

Three types of autophagy are known in plants, namely microautophagy, macroautophagy and mega-autophagy, with macroautophagy being the most well-characterized form of autophagy. In microautophagy, the vacuole directly engulfs cytoplasmic materials through the invagination of the tonoplast. Macroautophagy relies on a double-membrane transport vesicle, called an autophagosome, which engulfs and delivers the cytoplasmic cargo to the vacuole to be degraded [4]. Macroautophagy can be non-selective, resulting in the bulk degradation of cytoplasmic components, or selective, resulting in the targeted degradation and recycling of specific components such as mitochondria (mitophagy), chloroplasts (chlorophagy), ribosomes (ribophagy), peroxisomes (pexophagy), proteasome (proteaphagy) and endoplasmic reticulum (ER) (ER-phagy) [5]. Mega-autophagy is an extreme and often terminal process linked to conditions such as senescence and pathogen attack, which induce programmed cell death. During mega-autophagy, the tonoplast breaks to release vacuolar hydrolases, which then degrade the cytoplasm [4].

During macroautophagy (hereafter termed autophagy), autophagosome initiation, formation and function are facilitated by the autophagy-related (ATG) proteins, which are highly conserved across eukaryotes and classified into four main core groups [2]. The ATG1/ATG13 kinase complex is triggered by stress to initiate the formation of a cup-shaped double-membrane structure called the phagophore, which begins to wrap around the cargo. The phosphatidylinositol 3-kinase (PI3K) complex promotes phagophore nucleation, while the ATG2/ATG9/ATG18 complex promotes phagophore expansion. The ATG conjugation machinery facilitates the maturation and completion of the autophagosome, which is then delivered to the vacuole [2]. Disrupting the function of ATG proteins in plants decreases resistance to different stresses [6–11], while overexpressing *ATG* genes yields the opposite effect [12–15].

Agronomic systems face the challenge of sustaining crop productivity and feeding people amid an increasing global population, diminishing resources and a changing climate. The critical roles that autophagy plays in plant development and in safeguarding plants against multiple stresses [16–18] make autophagy a promising target to improve crop survival and productivity in suboptimal conditions (figure 1). There has been recent progress in elucidating the mechanisms that regulate the autophagy process in plants [2], providing an avenue to identify factors that can be manipulated to provide agronomic benefits. In this review, we discuss the function of autophagy in response to various abiotic stresses, highlighting the significance of strategically targeting key genes within the autophagy pathway for plant stress management. We also use publicly accessible gene expression data to evaluate the potential of targeting autophagy for improved tolerance of multifactorial stress conditions. For more details on selective autophagy mechanisms and receptors and on autophagy regulation in plants, we refer the reader to the following reviews [2,19–22].

## 2. Role of autophagy in abiotic stress tolerance

### (a) Nutrient stress

Crops require 17 essential nutrients (carbon, hydrogen, oxygen, boron, chlorine, copper, iron, manganese, molybdenum, nickel, zinc, nitrogen, phosphorus, potassium, sulfur, calcium and magnesium) for proper growth and development [23]. Imbalances, whether deficiencies or excesses, in these nutrients are common abiotic stresses that can negatively impact crop yield and quality [24]. The role of autophagy in plant response to nutrient stress, especially carbon and nitrogen starvation, is extensively documented.

In Arabidopsis (*Arabidopsis thaliana*), disrupting autophagy decreases tolerance of carbon and nitrogen starvation [7,10,25,26]. Under carbon starvation, autophagy-defective mutants exhibit delayed growth, decreased amino acid levels, increased respiration, decreased net protein biosynthesis and impaired lipid degradation [27]. Under nitrogen starvation, autophagy-defective mutants show premature leaf senescence and decreased nitrogen use and remobilization efficiencies [28], along with increased accumulation of ammonium, amino acids and proteins and a significant reduction in sugar levels [29]. These findings suggest that autophagy plays a critical role in maintaining metabolic balance and promoting survival during carbon and nitrogen deficiencies. The positive role of autophagy in carbon and nitrogen starvation tolerance was also shown in other plant species such as apple (*Malus domestica*) [12,14,30,31], rice (*Oryza sativa*) [32,33], maize (*Zea mays*) [34–36], tomato (*Solanum lycopersicum*) [37], foxtail millet (*Setaria italica* L.) [38] and the tea plant (*Camellia sinensis*) [39]. These studies show that overexpressing or disrupting specific *ATG* genes results in altered responses to carbon or nitrogen deficiency. Furthermore, the functions of regulatory proteins that modulate autophagy in plants at multiple regulatory levels in response to carbon or nitrogen deprivation are being elucidated, adding to the list of genes that could be targeted to enhance starvation tolerance in an autophagy-dependent manner [2,40,41].

Phosphorus, a macronutrient that plants take up primarily as inorganic phosphate (Pi), is essential for plant growth but is often present in limited and not readily accessible amounts in the environment [42]. A functional autophagy pathway is required to maintain phosphate homeostasis and promote plant fitness and primary root growth during phosphate shortage [43,44]. Autophagy also stabilizes the steady-state protein levels of the phosphate transporter 1 (PHT1) family, which participates in Pi uptake and remobilization under Pi-sufficient and Pi-deficient conditions [43]. Moreover, chlorophagy induced by carbon/nitrogen imbalance promotes Pi starvation tolerance in Arabidopsis, indicating a critical role for autophagy in coordinating plant responses to different nutrient stresses [45]. Under sulfur deficiency, disrupting autophagy results in accelerated leaf chlorosis, decreased plant biomass, increased metabolite accumulation, and impaired remobilization of sulfur from the rosettes to the seeds [46]. Similarly, defects in autophagy hamper the distribution of iron in Arabidopsis and hinder the translocation of iron, zinc and manganese to seeds [47]. In response to zinc deficiency, autophagy is induced to prevent leaf damage and death and alleviate light- and iron-mediated oxidative stress [48]. Autophagy is also activated by excess zinc stress and is required for the optimal distribution of iron in the plant to prevent excess zinc-induced iron starvation [48]. In grapevine (*Vitis vinifera*), high copper stress induces *ATG* gene expression and increases autophagosome formation in a time-dependent manner [49]. Recently, *VvATG6* overexpression was shown to promote reactive oxygen species (ROS) homeostasis and confer enhanced tolerance to copper stress in grape [50]. These observations collectively highlight the significance of a functional autophagy pathway in plant tolerance and resistance during fluctuations in nutrient availability.

In summary, autophagy is a vital cellular mechanism that enhances nutrient stress tolerance by facilitating nutrient recycling, remobilization and distribution to promote cellular homeostasis and energy conservation. Targeting autophagy through genetic manipulation or regulatory modulation can significantly improve the ability of plants and crops to withstand stresses induced by nutrient deficiencies or excesses.

### (b) Salt stress

Salt stress occurs when excessive salt accumulates in the soil and it poses a major environmental challenge that affects plant growth and productivity. High soil salinity disrupts water uptake, causing dehydration and ionic imbalances, which impair various physiological processes including photosynthesis, nutrient absorption and cellular metabolism. Consequently, plants often experience stunted growth, reduced yields and increased vulnerability to diseases [51–53]. To cope with salt stress, plants employ various strategies such as adjusting osmotic pressure, sequestering ions and synthesizing protective substances [51]. Nonetheless, severe or prolonged salt stress can surpass these adaptive responses, underscoring the need to develop improved salt-tolerant plant varieties.

In Arabidopsis, autophagy is activated in response to high salt conditions [8,54]. Plants with impaired autophagy have increased sensitivity to salt stress compared with wild-type plants [8], including during germination [54], highlighting the importance of autophagy in managing these environmental stresses. Furthermore, salt treatment activates the expression of *ATG* genes in various species including Arabidopsis [8], poplar (*Populus alba × P. tremula var. glandulosa*) [55], maize [56] and wheat (*Triticum* spp.) [57]. Moreover, overexpressing *ZmATG3* in Arabidopsis [56], *PagATG18a* in poplar [55] and *MdATG18a* in apple [58] improves salt tolerance by enhancing antioxidant activities during salt stress, implying an essential role for autophagy in plant salt stress tolerance. In addition, Arabidopsis plants overexpressing *CsATG18a* and *CsATG18b*, from sweet orange (*Citrus sinensis*), display significantly improved germination rates under salt stress conditions compared with the wild-type [59]. Spermidine, a type of polyamine, enhances salt tolerance in cucumber (*Cucumis sativus* L. cv. Jinchun No. 4) by activating *ATG* gene expression and promoting autophagosome formation under salt stress conditions. Silencing the *ATG4* and *ATG7* genes diminishes the salt tolerance and autophagosome formation induced by spermidine in cucumber [60]. This underscores the significant role of autophagy in enhancing plant resilience to salt stress.

Next to BRCA1 gene 1 (NBR1), a selective autophagy receptor, plays an important role in salt tolerance in Arabidopsis by interacting with ATG8 and targeting ubiquitinated aggregates of proteins for autophagic degradation [61]. *nbr1* mutant plants of Arabidopsis [61] and rice [62] are hypersensitive to salt stress, phenocopying autophagy-deficient mutants such as *atg5* and *atg7*. Conversely, overexpressing *OsNBR1* in rice [62] and *PagNBR1* in poplar resulted in increased salt tolerance [63]. In rice, *nbr1* mutants show increased ROS production and accumulation, whereas *OsNBR1* overexpression lines show reduced ROS production relative to wild-type plants [62]. Analogously, overexpressing *PagNBR1* in poplar increases the activity of antioxidant systems that are essential to regulate cellular homeostasis [63]. These results suggest that *NBR1* confers salt stress tolerance by regulating the levels of ROS in plants to prevent salt stress-induced oxidative damage.

Ufmylation is a post-translational modification in which the ubiquitin-like protein ubiquitin-fold modifier 1 (UFM1) is attached to proteins. Ufmylation is triggered by a range of stress conditions and requires the E3 ligase UFM1-protein ligase 1 (UFL1), which catalyses the covalent attachment of UFM1 to lysine residues on target substrates [64]. Salt stress triggers the interaction of UFL1 with ATG1, ATG6 and ATG8 proteins and induces its association with the ER and autophagosomes [65]. These interactions facilitate the selective turnover of dysfunctional ER via the autophagy pathway, thereby maintaining ER homeostasis [65,66]. ER proteotoxic stress, caused by ribosomal stalling, induces the formation and activation of a tripartite receptor complex involving UFL1, its membrane adaptor DDRGK1 and the ATG8-interacting protein C53 [67]. The activated complex facilitates ribosome-associated quality control via the autophagy pathway to maintain proteostasis during ER stress [67]. The Arabidopsis *ufl1* mutant has higher sensitivity to salt and ER stresses than wild-type plants, indicating a significant role for ufmylation in salt and ER stress tolerance [65,67]. Under salt stress, the loss of UFL1 function disrupted the effective degradation of damaged ER through ER-phagy, resulting in the accumulation of large ER sheets [65]. This implicates ufmylation in regulating ER-phagy and ER homeostasis during salt stress-induced ER damage.

### (c) Drought stress

Drought stress is a major environmental challenge that can lead to significant reductions in agricultural yields [68]. From agricultural and physiological perspectives, drought stress can be defined as the negative effects on plants owing to prolonged periods of insufficient water availability in the soil [69,70]. Drought conditions lead to ROS accumulation that can damage cell membranes, enzymes and other proteins. This leads to photosynthesis inhibition and metabolic disruption that ultimately limit plant growth and development [71–73].

Autophagy enhances plant tolerance to drought. Drought stress leads to the increased activation of *ATG* genes in Arabidopsis [8] and in several crops including cereals [38,74–80], fruits [13,14,59,81–84] and vegetables [15,85–87]. Arabidopsis lines with RNAi-mediated knockdown of *ATG18a* show reduced formation of autophagosomes and increased sensitivity to both drought and mannitol-induced osmotic stress [8]. Overexpression of *MdATG5a* [81], *MdATG10* [88] and *MdATG18a* [13] in apples improves drought tolerance, resulting in greater photosynthetic efficiency, reduced ROS levels, increased water content, increased autophagosome formation and elevated expression of *ATG* genes compared with wild-type plants. Transgenic Arabidopsis lines overexpressing *SiATG8a* from foxtail millet [76], *MdATG3a* and *MdATG3b* from apple [14], *MaATG8f* from banana (*Musa acuminata*) [82] and *CsATG18a* from sweet orange [59] exhibit improved tolerance under drought stress. In wild emmer wheat (*Triticum turgidum* ssp. *dicoccoides*), *TdATG8* silencing impairs drought tolerance accompanied by increased oxidative stress [75]. *MaATG8f* overexpressing lines display increased abscisic acid (ABA) sensitivity and increased levels of endogenous ABA [82], a phytohormone that promotes responses such as stomatal closure to reduce water loss under drought [89].

Autophagy facilitates the removal of oxidized and aggregated proteins under various stress conditions, including drought stress [11,90]. Under drought, autophagy facilitates the breakdown of aquaporins to minimize water loss [91]. Autophagy also modulates brassinosteroid (BR) signalling to promote plant survival under drought by selectively degrading brassinosteroid-insensitive 1 (BRI1)-EMS-suppressor 1 (BES1), a positive regulator of BR signalling and a negative regulator of drought tolerance [92,93]. Additionally, autophagy is activated under drought to selectively degrade constitutively stressed 1 (COST1), a negative regulator of both autophagy and drought tolerance [94]. The targeted degradation of both BES1 and COST1 by autophagy likely shifts plants from a growth phase to a drought response phase, thereby enhancing tolerance to drought.

The class A heat shock factor 1A (HSFA1A) activates *ATG* gene expression and autophagy in tomato to remove drought-induced insoluble protein aggregates, thereby increasing drought resistance [86]. Similarly, the ethylene response factor 5 (ERF5) transcriptionally activates autophagy to promote ethylene-dependent drought resistance in tomato [87]. The level of autophagy activity closely correlates with the activity of antioxidant systems under drought [81,82]. In tomato, ROS signalling mediated by the mitochondrial alternative oxidase (AOX) is crucial for triggering autophagy to promote ethylene-mediated drought tolerance [87]. This suggests that autophagy is required to attenuate drought stress-induced oxidative stress in plants. Collectively, these findings demonstrate that autophagy activation is integrated into several drought response pathways to enhance plant tolerance.

### (d) Heat stress

Plants require an optimum temperature for their growth and development. High temperature can cause protein misfolding, denaturation and aggregation, resulting in the loss of membrane integrity and metabolic imbalance [15,95,96]. This ultimately results in retarded plant growth and reduced yield [97–99]. Moreover, heat stress (HS) can lead to other stresses such as oxidative stress [96,100,101] and ER stress [102] owing to overproduction of ROS and protein misfolding, respectively.

Autophagy plays a crucial role in plant thermotolerance by removing and recycling misfolded or denatured proteins and maintaining cellular homeostasis under HS [103]. Autophagic activity increases in response to HS in Arabidopsis [61] and in crop species such as pepper [15], tomato [100,103,104] and apple [105]. Disrupting autophagy in Arabidopsis causes hypersensitivity to HS during the reproductive stage, ultimately leading to male sterility [106]. Similarly, silencing autophagy impairs heat tolerance in tomato plants [103]. In apple, *MdATG18a* overexpression results in improved heat tolerance [105], and in tomato, *SlATG8f* overexpression enhances pollen viability under HS [104]. HSFA1A, a positive regulator of HS responses, directly activates *ATG10* expression and promotes autophagy in tomato to degrade heat-induced ubiquitinated proteins. This contributes to pollen viability and thermotolerance under HS [107]. Disrupting the function of WRKY33, another key regulator of thermotolerance [108], decreases the expression of *ATG* and *NBR1* genes, resulting in impaired autophagy and decreased tolerance of HS [61,103].

Thermomemory refers to the ability of plants to maintain thermotolerance after an initial exposure to mild HS, allowing an efficient response to future HS events [109]. Resetting thermomemory requires the degradation of heat shock proteins (HSPs) during the recovery period [110–114]. Thermopriming, defined as the pre-conditioning of plants with elevated but non-lethal temperatures, induces autophagy [113,115]. Following thermopriming, autophagy activity remains high during the recovery phase and selectively degrades HSPs, thereby resetting thermomemory in the primed plant [113,115]. As a result, mutants with impaired autophagy retain HSPs for longer periods, leading to better tolerance during subsequent HS than wild-type plants [113,115]. In summary, these studies demonstrated that disrupting autophagy (i) severely decreases basal thermotolerance, (ii) has no impact on acquired thermotolerance, and (iii) improves thermomemory in Arabidopsis [113,115].

During heat-induced ER stress, Arabidopsis inositol requiring 1-1 (IRE1B), a serine/threonine-protein kinase and endoribonuclease, is activated and splices the mRNA of the transcription factor basic region/leucine zipper motif 60 (bZIP60). This leads to the production of a spliced, active form of bZIP60, which translocates to the nucleus and promotes the transcription of genes, such as binding protein 3 (BIP3), involved in the unfolded protein response [116]. Heat-induced ER stress activates autophagy in an IRE1B- and unfolded protein-dependent manner in Arabidopsis [117,118]. The ER is degraded via autophagy during ER stress, and the delivery of ER to the vacuole is also dependent on the function of IRE1B [117]. Moreover, autophagy is critical for the survival of plants under ER stress [118]. These findings demonstrate the importance of autophagy under ER stress and the critical role of IRE1B in this process.

### (e) Cold stress

Cold stress, which occurs at chilling (0–15°C) and freezing (below 0°C) temperatures, negatively impacts plant growth and survival [119]. Compared with other stresses, studies into the role of autophagy in cold stress tolerance are relatively few and recent. Overexpression of alfalfa (*Medicago sativa* L.) *MsATG13* gene in transgenic tobacco (*Nicotiana tabacum*) enhances tolerance to chilling stress by promoting autophagosome formation, upregulating *ATG* gene expression and increasing antioxidant activity and proline content to counteract cold-induced oxidative stress [120]. In Arabidopsis, overexpression of the enzyme phospholipid:diacylglycerol acyltransferase 1 (PADT1) boosts plant fitness under chilling stress accompanied by increased autophagic activity, increased plant biomass and increased seed yield, weight and acyl lipid content [121]. Heterologous overexpression of *CsATG18b* protects Arabidopsis leaves against freezing-induced damage [59]. Autophagy can therefore be targeted to boost plant resistance under cold temperatures.

In tomato, brassinazole-resistant 1 (BZR1), a positive regulator of BR signalling [122], promotes BR- and autophagy-mediated tolerance of chilling stress through the transcriptional induction of *NBR1* and *ATG* genes [123]. BZR1 also enhances survival of Arabidopsis plants at freezing temperatures by inducing the expression of the cold-responsive *C-repeat binding factor* (*CBF*) genes [124]. These findings indicate that BZR1-mediated BR signalling promotes cold stress tolerance through various pathways, making the BR signalling pathway a good candidate to target for plant resilience under low temperatures. In Arabidopsis, short-term BR treatment reduces target of rapamycin (TOR) activity (a negative autophagy regulator) and increases autophagy activity, facilitating cellular remodelling [125,126]. However, under prolonged BR treatment, TOR activity is restored and autophagy is suppressed, likely conserving cellular resources for other growth processes [125]. brassinosteroid-insensitive 2 (BIN2), which negatively regulates BR signalling by destabilizing BZR1 and its paralog BES1 [93,122], promotes autophagy activity in Arabidopsis [125] but represses BZR1/BES1-mediated tolerance of Arabidopsis to freezing stress [124]. In addition, BZR1 and BES1 are targets of autophagic degradation in Arabidopsis [93,127]. Recent reports show that autophagy activity in Arabidopsis is suppressed under cold treatment [121,128], and a functional autophagy pathway is dispensable for tolerance of Arabidopsis plants to freezing temperatures [128]. The results from these different studies suggest that autophagy may be suppressed in a BR-dependent manner to promote BZR1/BES1-mediated freezing tolerance in Arabidopsis. Collectively, these studies indicate that targeting autophagy to improve cold stress tolerance may be dependent on (i) plant species, (ii) type of cold stress (whether chilling stress or freezing stress), and (iii) type of autophagy (whether selective or non-selective).

### (f) Hypoxia

Hypoxia is a stressful and severe condition that occurs when there is a decline in oxygen availability, thereby causing the oxygen level to be insufficient for aerobic respiration [129]. Hypoxia can impact crop productivity and survival and arises from adverse conditions such as flooding-induced submergence and waterlogging [129]. Submergence takes place when the plant is completely or partially (roots and some portion of the shoot) immersed in water. In contrast, waterlogging occurs when only the root portion of the plant is immersed in water owing to water saturation of the soil [129,130]. Besides oxygen deprivation, hypoxic conditions can trigger multiple stresses in plants including low light, nutrient and energy deficiency, osmotic stress, salinity, accumulation of toxins and oxidative stress following reoxygenation [131,132]. This makes hypoxia a significant environmental challenge for plant health and survival.

Submergence-induced hypoxia activates autophagosome formation and increases autophagic activity in Arabidopsis. Autophagic flux is proportional to the duration of submergence, i.e. the longer the submergence, the higher the autophagy activity [6,133]. The severity of hypoxia increases as it progresses, which likely elevates cellular damage in a cumulative manner. As a result, autophagy activity may increase in proportion to the severity of the stress-induced damage. Submergence induces the expression of several key *ATG* genes [6,133]. A similar *ATG* gene expression profile is also observed in Arabidopsis roots during waterlogging stress [134]. The sustained expression of these *ATG* genes [6,133,134] likely replenishes the autophagy machinery to maintain autophagy activity during hypoxia. Loss of autophagy alters the expression of critical hypoxia- and ethylene-responsive genes, which regulate hypoxia signalling in plants. Several of these genes are downregulated in autophagy-defective mutants under submergence [6]. In addition, disrupting autophagy decreases the tolerance of Arabidopsis to submergence stress with significantly reduced plant biomass and survival [6,133]. This suggests that a functional autophagy pathway is required to execute a proper response to submergence stress.

A defective autophagy pathway increases the expression of genes involved in ROS generation and downregulates genes involved in ROS scavenging activities [6]. This misregulation impairs the function of the cell’s antioxidant system, leading to excess ROS accumulation under submergence and leaving the plant defenceless against oxidative stress. ROS itself plays a key role in activating autophagy under hypoxia, as ROS-deficient mutants display impaired autophagic response and decreased submergence tolerance [6]. These findings suggest that a certain threshold of ROS level is required to promote autophagy activation, and in return, autophagy maintains ROS homeostasis to prevent oxidative stress-induced cellular damage during hypoxia. In comparison, waterlogging induces similar ROS-dependent autophagy induction in Arabidopsis roots to promote root cell viability and attenuate hypoxia-induced programmed cell death [134]. Another way in which autophagy maintains ROS homeostasis during hypoxia is by regulating salicylic acid (SA) signalling. Autophagy deficiency promotes SA synthesis and signalling, which enhances ROS accumulation in autophagy-defective mutants under submergence [6]. Interestingly, SA signalling is required for submergence-induced autophagy activation [6]. These findings suggest that SA signalling induces autophagy under hypoxia, and in return, autophagy modulates the SA signalling pathway to prevent oxidative stress-induced damage.

Hypoxia causes plant cells to switch to anaerobic fermentation to generate energy, and this involves the lactic acid and ethanolic fermentation pathways. During ethanolic fermentation, pyruvate decarboxylase and alcohol dehydrogenase (ADH) coordinate to convert pyruvate (a product of glycolysis) to ethanol, regenerating nicotinamide adenine dinucleotide (NAD^+^) for continued glycolysis and ATP production [135]. Ethanol production in plants during anaerobic respiration is generally harmless owing to its rapid diffusion out of cells, except at high concentrations where it can impact plant growth [135–137]. Exogenous ethanol treatment induces *ATG* gene expression and enhances autophagy activity in Arabidopsis. Autophagy-defective mutants are intolerant of high concentrations of ethanol and display impaired growth, reduced chlorophyll content, increased chlorosis and increased cell death under such conditions [6,137]. In addition, disrupting endogenous ethanol production by knocking out *ADH1* represses autophagosome formation during submergence and causes hypersensitivity to submergence stress [137]. These results suggest that the production of ethanol during hypoxia likely facilitates autophagy activation that further promotes tolerance of submergence stress. Autophagy is also induced by hydrogen sulfide (H_2_S) during submergence. Under normoxia, H_2_S suppresses *ATG* gene expression and autophagy to maintain a low basal level of autophagy activity [133,138]. Upon submergence-induced hypoxia, H_2_S induces *ATG* gene expression and increases autophagy activity to promote submergence tolerance in Arabidopsis [133,138]. Autophagy also contributes significantly to seed germination under hypoxia. Hypoxia induces nitric oxide (NO) accumulation and signalling that facilitates the S-nitrosylation of S-nitrosoglutathione reductase 1 (GSNOR1), causing the protein to be selectively degraded by autophagy. These events result in a feedback loop that further promotes NO signalling to prompt seed germination in Arabidopsis [139]. Overall, these observations further emphasize the critical role of autophagy in mediating various responses to submergence stress.

Hypoxia triggers the formation of cytoplasmic stress granules, which are transient structures that sequester non-essential mRNAs and translation initiation factors or messenger ribonucleoprotein complexes. Stress granules help the cell to effectively pause translation, reduce ATP utilization and conserve cellular resources for processes involved in hypoxia resistance and adaptation [140–144]. Autophagy facilitates the clearance of hypoxia-induced stress granules [145–147]. These stress granules accumulate in the early stages of hypoxia in plants but are degraded by autophagy as hypoxia progresses [145]. This may help the cell to clear and recycle non-essential or damaged proteins and RNA for use as additional energy sources and provide a means for cellular resources to be used for repair, survival, and adaptation.

### (g) Light stress

Sunlight is the main energy source for photosynthesis and plays a key role in plant growth and development. Plants experience different light intensities in the environment, which can induce stress and affect their photosynthetic efficiency [148,149]. Light stress can be caused by low light, high light (HL) and ultraviolet-B (UV-B) radiation. The severity of light stress and the corresponding physiological responses are determined by the fluence rate and duration of the stress. To withstand light stress, plants must balance their energy requirements with light intensity while simultaneously resolving any light-induced cellular damage.

Low light diminishes the amount of energy accessible for photosynthesis and normal plant function and it can be caused by factors like dense canopy cover, seasonal changes that shorten daylight hours, and persistent cloudiness [150–154]. Starchless Arabidopsis mutants mimic limited or low energy status induced in plants by low light conditions. Under shortened photoperiods, both starchless and autophagy-deficient mutants display similar rates of growth retardation. Disrupting autophagy in starchless mutants exacerbates this phenotype, accompanied by significantly reduced shoot biomass and accelerated leaf cell death [155]. Moreover, autophagy deficiency compromises the availability of other possible sources of energy in a starchless mutant, likely owing to the loss of autophagy-dependent catabolism of amino acids and nucleotides [155,156]. Together, these observations strongly indicate that autophagy significantly contributes to plant growth and adaptation to energy fluctuation induced by limited light availability.

Low blue light (LB) and a low red-to-far-red ratio are key light signals associated with vegetative shade, and they play crucial roles in triggering the shade avoidance syndrome in plants. This adaptive response involves several growth adjustments, including hypocotyl elongation and increased petiole length, aimed at maximizing light capture to support photosynthesis [157–160]. LB activates autophagy, and autophagy-defective mutants exhibit impaired hypocotyl elongation in response to LB [161]. These results indicate that autophagy can be activated in plants under vegetative shade in an LB-dependent manner, likely resulting in nutrient recycling and distribution for shade avoidance-related growth.

Excess photosynthetically active radiation (400–700 nm), which results in HL stress, and excess UV-B radiation (280–315 nm) from the sun cause light-induced damage in plants. HL and UV-B decrease the photosynthetic capacity of plants through photoinhibition, a light-induced inactivation of photosystem II, with a reduction in chlorophyll content and Rubisco activity. HL and UV-B also generate excess ROS, leading to photooxidative stress, which can damage DNA, proteins, lipids, cell membranes and organelles such as chloroplasts, mitochondria and peroxisomes [148,149]. HL induces abnormal chloroplast swelling, triggering the formation of ATG8-labelled membrane structures that facilitate the tonoplast-mediated engulfment and vacuolar degradation of the damaged chloroplasts [162]. This process resembles microautophagy and is dependent on the core autophagy machinery [162]. The autophagy receptor NBR1 associates with a distinct sub-population of damaged chloroplasts during HL stress and mediates their removal from the cytoplasm in a microautophagy-like manner that is independent of the core ATG machinery [163]. The selective targeting of photodamaged chloroplasts in this manner may provide plant cells with flexibility in responding to varying degrees of damage and optimizing chloroplast quality control under HL stress. The core ATG machinery is also required for the formation of ATG8-/ATG18a-decorated membrane structures that mediate the selective targeting and degradation of HL-induced peroxisome aggregates [164]. This mitigates ROS-mediated oxidative damage, thereby promoting leaf health under HL stress [164].

Autophagy-defective mutants exposed to UV-B display a significant decrease in growth, biomass, photosynthetic efficiency and survival, accompanied by an increase in cell death, ROS and cyclobutane pyrimidine dimer levels relative to wild-type plants [165–167]. Autophagy removes UV-B-damaged chloroplasts by means of tubular-shaped autophagosomal structures, which sequester the chloroplasts from the cytoplasm and deliver them to the vacuole for degradation [166]. Autophagy also facilitates mitochondria quality control in UV-B-exposed leaves by clearing fragmented and depolarized mitochondria from the cytoplasm [165,167]. These results demonstrate that autophagy is activated in HL- and UV-B-exposed leaves and prevents leaf cell death by facilitating the selective clearance of photodamaged chloroplasts, mitochondria and peroxisomes. A functional autophagy process is therefore required to promote plant growth and survival under light stress-induced damage.

## 3. Combined stresses

The role of autophagy in plant resilience to individual stresses is well documented [3,16]. However, in natural environments, plants often encounter multiple stressors simultaneously (referred to as combined stresses), which can have complex and sometimes synergistic effects on plant growth, development and survival [168]. At present, there is a limited body of research on the relationship between autophagy and combined stresses, highlighting a significant gap that requires further studies.

**Figure 2.**
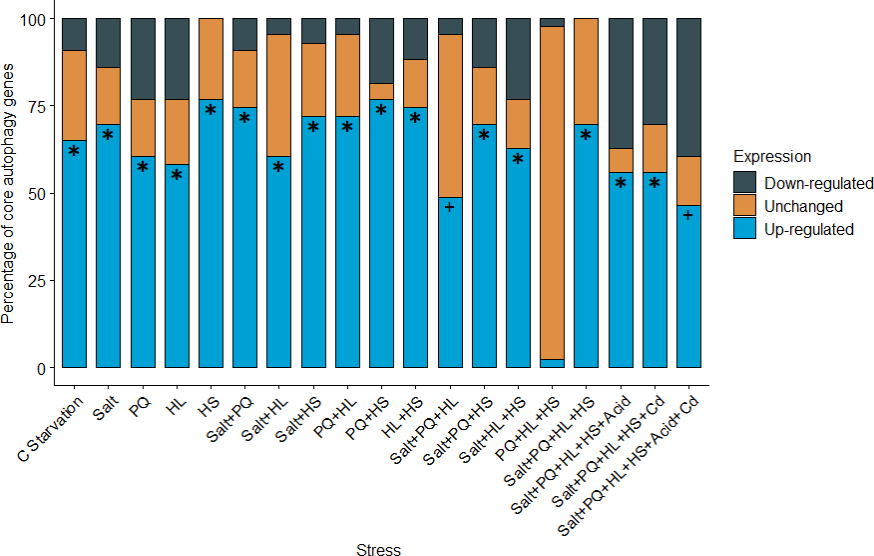
Stacked bar plot showing percentages of 43 core autophagy-related (*ATG*) genes that are upregulated, downregulated or remain unchanged in the indicated stress condition from GSE93420 (C starvation) [93,170] and GSE147962 (all other stresses) [169] expression datasets. Asterisks (∗) indicate stress conditions in which >50% of *ATG* genes are significantly upregulated. Plus sign (+) indicates stress conditions in which 25–50% of *ATG* genes are significantly upregulated. C starvation, carbon starvation; Cd, cadmium; HL, high light; HS, heat stress; PQ, Paraquat.

Arabidopsis possesses 43 core *ATG* genes, including their respective isoforms, with the ATG1 complex, ATG9 complex, PI3K complex and ATG8 conjugation systems consisting of 8, 11, 6 and 18 genes, respectively [41]. The degree of induction of the core *ATG* genes under different multifactorial stresses can offer insights into the level of autophagy activity in plants during these conditions. We retrieved and analysed *ATG* gene expression profiles from publicly available transcriptomic datasets [93,169,170] and found that single stresses such as carbon starvation (C starvation), NaCl-induced salt stress (salt), paraquat (PQ)-induced oxidative stress, HL and HS upregulate more than 50% of the core *ATG* genes (figure 2). This is not surprising, as these individual stresses significantly increase autophagy activity in plants [10,11,54,61,103,163,164]. While most stress combinations significantly upregulated *ATG* genes, salt + PQ + HL, PQ + HL + HS and salt + PQ + HL + HS did not seem to activate *ATG* gene expression to a similar degree as the other combined stresses (electronic supplementary material, figure S1A). These results indicate that the effects of combined stress on *ATG* gene expression and likely autophagy activation are not simply additive, and the activation of alternative pathways may be compensating to manage these stresses. It will be interesting to verify this through functional studies.

It should also be noted that the downregulation of some *ATG* genes under combined stresses (figure 2) is likely required to regulate the intensity of the autophagy process to prevent excessive autophagy, which could lead to cell death [171–173]. LUX ARRYTHMO (LUX) and timing of CAB expression 1 (TOC1) are circadian clock transcription factors that repress specific *ATG* genes under nutrient deficiencies to moderate the levels of autophagy activity and promote stress tolerance [171,173]. *LUX* and *TOC1* exhibit similar expression patterns under single and combined stresses (electronic supplementary material, figure S2A) and are proposed to work together to modulate autophagy levels [2]. It will be interesting to know whether LUX and TOC1 perform similar regulatory functions under other stresses in addition to nutrient starvation. ATAF1 and WRKY33 are positive transcriptional regulators of *ATG* gene expression in Arabidopsis [103,174]. The expression patterns of *ATAF1* and *WRKY33* correlated with that of *ATG* genes under most stress conditions (electronic supplementary material, figures S1 and S2), suggesting that these factors may modulate *ATG* gene expression and autophagy under these conditions. Additional research is required to validate this hypothesis.

Recent studies show that the loss of autophagy compromises survival, leaf health, photosynthetic efficiency and stomatal function under HL + HS conditions compared with HL or HS single stresses [175]. A similar phenotype was also observed in mutants deficient in the synthesis of gamma-aminobutyric acid (GABA) [175], a non‐proteinogenic amino acid implicated in autophagy and stomatal response regulation [3,176–179]. These data suggest that GABA and autophagy are essential for plant tolerance to combined HL + HS stresses.

In summary, available functional studies and transcriptomic data strongly suggest that autophagy is an important stress response pathway for plants under multifactorial stresses. Further studies are, however, required to confirm whether the changes in *ATG* gene expression correlate with autophagy activity at the cellular level and whether this directly translates to improved resilience in plants under different combined stresses.

## 4. Targeting autophagy for improved stress resilience

As global climate change accelerates, there is an urgent need to develop crops that can withstand multiple environmental stressors. As a crucial stress response pathway, autophagy is a promising target for improving crop stress resilience. A common approach to enhance autophagy is to genetically engineer crops to overexpress key *ATG* genes or genes involved in autophagy regulation, which confer increased plant tolerance to environmental stresses (table 1). The overexpression of these target genes can enhance autophagy and improve several parameters needed for stress tolerance, including plant growth and biomass, photosynthetic efficiency, antioxidant activities and even water retention in the case of heat and drought stresses (table 1). To fine-tune autophagy activity, crops could be engineered to co-express a key *ATG* gene along with a negative regulator, such as *LUX* or *TOC1*, which modulates autophagy intensity under stress. Such moderator genes control autophagy levels to prevent excessive autophagy-induced cell death. Targeting autophagy in this manner may aid in optimizing autophagy and stress tolerance in crops without compromising plant health, development or yield. Finding additional moderator genes and elucidating their roles (and the specific conditions in which they function) in the autophagy regulatory network can be effective in the manipulation of autophagy in crops.

**Table 1.** Examples of autophagy-related genes that improve abiotic stress tolerance when overexpressed.

species	gene	stress	improved parameters	references
Alfalfa	*MsATG13*	chilling	antioxidant activity, cell viability	[120]
Apple	*MdATG3a, MdATG3b*	–C^a^, –N^b^, salt, osmotic, drought	biomass, root growth	[14]
	*MdATG5a*	drought	cell viability, antioxidant activity, MDA^c^ content, water content, photosynthetic capacity, chlorophyll content, amino acid level	[81]
	*MdATG5a*	oxidative	chlorophyll content	[81]
	*MdATG7b*	–C, –N	biomass, plant growth	[31]
	*MdATG8i*	–C, –N	biomass, root growth	[30]
		salt	biomass, plant height, cell viability, chlorophyll content, MDA content	[180]
		drought	antioxidant activity, root viability, root water uptake	[181]
	*MdATG9*	–N	biomass, free amino acid levels, soluble sugar levels, nitrate assimilation	[182]
	*MdATG10*	drought	plant growth and biomass, cell viability, antioxidant activity, MDA content, photosynthetic efficiency, chlorophyll content, water content, water use efficiency	[88]
	*MdATG18a*	–N	biomass, root growth, anthocyanin content, nitrate assimilation	[12]
		drought	antioxidant activity, photosynthetic efficiency, water content, cell viability	[13]
		heat	water content, antioxidant activity, photosynthetic efficiency	[105]
		alkaline	GABA content, antioxidant activity, photosynthetic efficiency, chlorophyll content	[176]
		salt	cell viability, chlorophyll content, antioxidant activity, root vitality, biomass, Na^+^ concentration	[58]
Arabidopsis	*AtATG5, AtATG7*	oxidative	chlorophyll content, yield	[183]
	*AtTGA9*	–C	survival	[184]
	*AtPADT1*	chilling	biomass, yield, acyl lipid content	[121]
Banana	*MaATG8f*	drought	antioxidant activity, photosynthetic efficiency, ABA levels, MDA content	[82]
Foxtail millet	*SiATG8a*	–N	plant growth, N content	[76]
		drought	survival, chlorophyll content, antioxidant activity, MDA content, proline content	[76]
Grape	*VvATG6*	high copper	plant growth, biomass, antioxidant activity	[50]
Maize	*ZmATG3*	–N, salt, osmotic	biomass, root growth, antioxidant activity, leaf growth	[56]
	*ZmNBR1*	drought	survival, cell viability, water content, MDA content, photosynthetic efficiency	[185]
Poplar	*PagATG18a*	salt	photosynthetic efficiency, antioxidant activity	[55]
	*PagNBR1*	salt	plant growth, antioxidant activity, water content, MDA content, Net CO_2_ assimilation, photosynthetic efficiency	[63]
Rice	*OsNBR1*	salt	survival, ROS level reduction	[62]
Soybean	*GmATG8c*	–C, –N	biomass, survival	[186]
Sweet orange	*CsATG18a, CsATG18b*	salt, osmotic	germination rate	[59]
	*CsATG18a*	drought	cell viability	[59]
	*CsATG18b*	freezing	cell viability	[59]
Tomato	*SlAOX1a*	drought	photosynthetic efficiency, water content, cell viability	[87]
	*SlATG8f*	heat	pollen viability, chlorophyll B content	[104]
	*SlBZR1*	–N	chlorophyll content	[37]
		chilling	cell viability, photosynthetic efficiency	[123]
	*SlHSFA1A*	drought	water content, insoluble protein	[86]

^a^ Carbon starvation

^b^ Nitrogen starvation

^c^ Malondialdehyde

**Figure 3.**
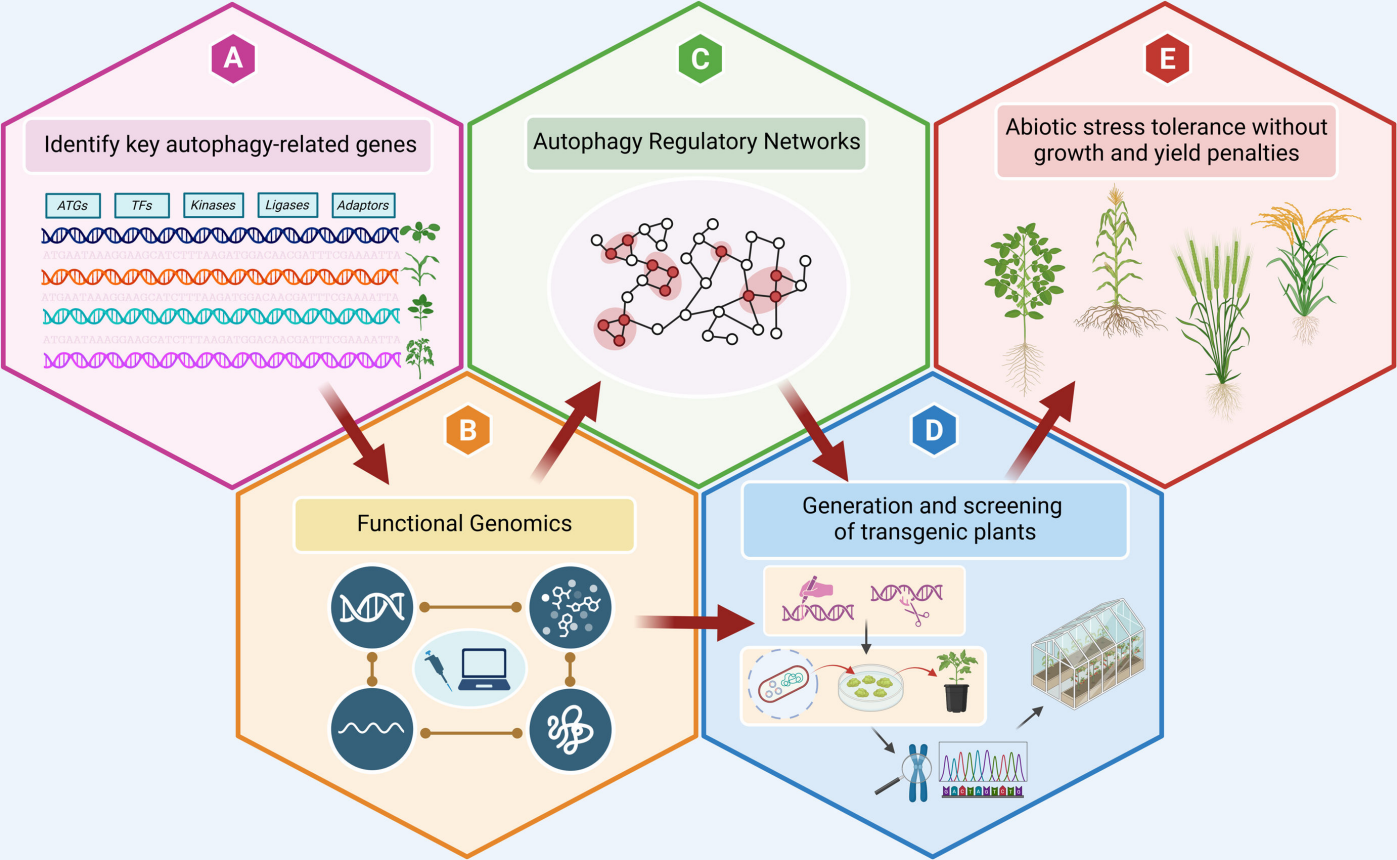
Strategic approach to enhance abiotic stress resilience in crops by targeting autophagy. (A) The process begins with the identification of key autophagy-related genes in extremophiles and/or selected crop species, providing a foundation for understanding stress responses. (B) Following gene identification, functional studies are conducted at multiple levels, including genome, transcriptome, proteome and metabolome, to elucidate the roles of these genes in stress responses. (C) A model of the autophagy regulatory network is then developed to integrate the findings and visualize interactions among key components. (D) This model guides the generation and screening of transgenic plants in crops of interest, aimed at enhancing their autophagy pathways. (E) This approach culminates in increased abiotic stress tolerance in these crops, achieved without adverse effects on growth or yield and thereby supporting sustainable agricultural practices.

At present, the regulatory mechanisms that govern plant autophagy in response to various stresses at different levels (epigenetic, transcriptional, post-transcriptional, post-translational) are not fully understood. A comprehensive understanding of such regulatory pathways is crucial for building a robust model of the autophagy regulatory network in plants (figure 3). Such a model can reveal how various environmental signals are integrated into autophagy activation and elucidate the roles of various factors in coordinating these events. A multidisciplinary approach comprising functional and omics (transcriptomics, proteomics and metabolomics) studies combined with network analysis will be instrumental in addressing this challenge (figure 3). A similar approach can be employed to understand the role of autophagy in multifactorial stress tolerance with a focus on (i) the effect of different combined stresses on autophagy activation; (ii) how autophagy influences changes in the transcriptome, proteome and metabolome in response to stress combinations; (iii) how these changes affect combined stress resilience in plants; and (iv) the interplay between autophagy and other stress signalling pathways under combined stresses. Such studies can be combined with research that investigates the response to multifactorial stress combinations of plant lines overexpressing *ATG* or autophagy regulatory genes. The data obtained from addressing these questions can offer critical insights and tools to precisely fine-tune plant responses to multiple stresses, enabling the development of crop varieties with enhanced resilience to climate change-induced environmental challenges.

Extremophiles thrive under extreme conditions such as high salinity, drought and extreme temperatures and they have often evolved unique and robust stress-responsive and adaptive mechanisms [187]. Identifying and characterizing key *ATG* genes from extremophiles, such as halophytes and drought-resistant plants, could provide valuable *ATG* gene variants that are naturally adapted to severe stress conditions. These gene variants can be heterologously overexpressed in a crop of interest and tested to confirm whether they provide the same protection against environmental stresses. However, it is essential to conduct thorough studies to ensure that the introduced gene does not compromise the growth, yield or any other agronomic traits of the target crop. Insights from how autophagy is regulated in these stress-resistant plants could be combined with CRISPR-Cas9-based technologies to introduce regulatory elements such as specific enhancers or regulons to the promoters of target crop *ATG* genes. This could allow crops to harness a more resilient autophagic response under stress. With advances in synthetic biology and protein engineering, transcription factors with domains that recruit specific chromatin modifiers or gene circuits coupled with ROS biosensors can also be designed to precisely enhance *ATG* gene expression in response to different stress conditions.

Certain metabolites and molecules have been shown to induce autophagy when applied exogenously, providing a non-transgenic route for autophagy induction. A recent study demonstrates that raffinose improves plant growth in an autophagy-dependent manner [188]. H_2_S is involved in various stress response pathways [189], and it activates autophagy under hypoxia when supplied exogenously [133]. Treatment with phytohormones such as BR induces autophagy in tomato [37,123] to improve stress tolerance. The key aspects of such applications are to establish the concentration, dosage and method of application required for autophagy induction and understand the various effects they may have on plant physiology after application.

## 5. Conclusions

Autophagy plays a pivotal role in plant stress tolerance by facilitating the degradation and recycling of cell components, thus maintaining cellular homeostasis during adverse conditions such as drought, nutrient deficiency, HS and salt stress. Through the ability to regulate energy balance, promote nutrient remobilization, facilitate organellar and protein quality control and sustain cell viability (figure 1), autophagy enhances the capacity of plants to withstand multiple environmental stresses. As a result, targeting autophagy for crop improvement presents a promising strategy, as it offers the potential to optimize stress responses at both molecular and cellular levels. By manipulating autophagy-related pathways, it may be possible to develop crops that exhibit enhanced resilience, better growth and increased yield under stress conditions (figure 3), ultimately contributing to agricultural sustainability in the face of global climate change.

## Data Availability

Gene expression analysis data was retrieved from GSE93420 [93,170] and GSE147962 [169] from Gene Expression Omnibus (GEO) Datasets. Supplementary material is available online [190].
